# Georgia Medical Association

**Published:** 1882-05-20

**Authors:** 


MEDICAL ASSOCIATION OF GEORGIA.—The Medical Association of Georgia met in the Senate Chamber, Atlanta, at 11 o'clock a. m., April 19th, 1882. Prayer was made by the Rev. H. H. Tucker, and the address of welcome was delivered by Dr. J. F. Alexander of this city, in very cordial terms, and responded to appropriately by Dr. Eugene Foster, of Augusta. The attendance was unusually large, and the opening evinced a revival of interest in the Association, and good feeling prevailed.

President Holt's address was upon the "Times, Difficulties and Responsibility of the Profession." The address was well conceived, ably written and calmly and gracefully delivered. He warmly urged the improvement of the Association and better attendance upon its meetings. He made eloquent reference to the assassination of President Garfield, and alluded in a touching manner to the death of five members of the Association since the last meeting, to wit: Drs. J. M. Green and Jno. R. Boon, of Macon; T. W. Grimes, of Columbus; J. W. Underwood, of Cave Springs; E. L. Crump, of Burke, and I. N. Vanmeter, of Bartow. He advocated the adoption of a law for the establishment of an Inebriate Asylum in Georgia, and that the Association memorialize the Legislature to that end.

Many papers of interest were presented, of which we have not space to particularize. Among these were the papers of Dr. Eugene Foster, on the Relative Merits of Humanized and Bovine Virus; Prof. Thos. S. Powell, on Gynecology; Dr. R. M. Brown, Hemorrhagic Malarial Fever; Prof. G. G. Roy, Suppurative Hepatitis; Reports of Census and on Gynecology, by Dr. Nunn, presenting instruments of his own invention.

More than the usual number of voluntary papers were read. Among these were presented on the first day, a paper on entropion and trichiasis, by Prof. A. G. Hobbs, and Prof. A. W. Calhoun; Dr. A. F. Scott, ergot in throat and lung affections, with cases, etc.; Dr. Philpot, on contagiousness of typhoid fever.

A committee appointed to consider the suggestion of the President, relative to memorializing the Legislature to establish an Inebriate Asylum, reported favorably.

A resolution in favor of a life membership for a single fee of $30, was adopted.

The Annual Oration was delivered by Dr. J. P. Stevens—a scientific address of much interest.

On the second day, Dr. Battey read an interesting paper in regard to his trip to the International Medical Congress.

Dr. Lallerstedt showed a case, in process of recovery, of a little girl whose entire scalp had been torn away by machinery.

A resolution by Dr. Baird, reaffirming the Code of Ethics, was unanimously adopted.

Papers were submitted by Drs. Love, O'Daniel, Jones, Bell, Hull, Prof. Thad. Johnson, and others, all of which will appear in the published Transactions.

The following are the officers nominated and elected for the ensuing year:

For President, Dr. K. P. Moore, of Forsyth; for first Vice-President, Dr. A. J. Whitehead, of Waynesboro; for second Vice-President, Dr. F. R. Calhoun; for Treasurer, Dr. Goodrich, of Augusta; for Censor, Dr. A. W. Calhoun, of Atlanta.

CHAIRMEN OF DISTRICT COMMITTEES:

1st. District—Dr. E. J. Charlton—Practice; Dr. J. D. Martin—Surgery.

2d. District—Dr. E. W. Alfriend—Practice; Dr. P. L. Helsman—Surgery; Dr. T. S. Hopkins—Gynecology.

3d. District—Dr. N. P. Jelks—Practice; Dr. A. A. Smith—Surgery; Dr. A. R. Taylor—Gynecology.

4th District—Dr. A. W. Griggs—Practice; Dr. A. B. Copeland—Surgery; Dr. T. J. Slaughter—Gynecology.

5th. District—Dr. W. G. Owens—Practice; Dr. Geo. G. Crawford—Surgery; Dr. J. L. Hamilton—Gynecology.

6th. District—Dr. C. Hall—Practice; Dr. F. H. Kennan—Surgery; Dr. W. H. Hall—Gynecology.

7th. District—Dr. W. B. Wells—Practice; Dr. C. P. Gordon—Surgery; Dr. J. C. Bevins—Gynecology.

8th. District—Dr. E. W. Hunter—Practice; Dr. D. Ford—Surgery; Dr. J. A. Eve—Gynecology.

9th. District—Dr. Hollingsworth—Practice; Dr. A. A. Bell—Surgery; Dr. McClesky—Gynecology.

Dr. Griggs introduced a resolution that the committee appointed to memorialize the Legislature on the subject of an Inebriate Asylum take an additional duty of presenting the matter of providing a law for the payment of witnesses called to give expert testimony. The resolution was amended so as to provide for a separate committee, and was passed.

EXPELLING A MEMBER.

Dr. Hall, of the Board of Censors, asked the Association if the Board had full authority in all cases where charges of a violation of the Code were preferred. A vote was taken, and the sense of the Association was in favor of giving the entire matter into the hands of the Board. The Board retired and soon returned with a resolution charging Dr. D. O. C. Heery, of Atlanta, with a violation of the Code of Ethics, by advertising as a specialist. Dr. Heery was declared expelled.

Dr. Kennan reported a case of fracture, made some remarks relative to the practicability in certain instances of treating fractures successfully without apparatus. Discussed by Drs. Crawford, Foster and Word, the latter claiming that the old starch apparatus, when judiciously used, was superior to any other in simple fractures of the extremities.

Dr. Bell made some remarks upon the hygienic value of circumcision. And other interesting topics were broached by members, which we have not space to publish.

Dr H. H. Battey, of Cartersville, was appointed Orator for the next meeting.

The following delegates to the meeting of the American Medical Association were appointed by the President.

J. P. Logan, W. F. Westmoreland, Henry F. Campbell, Robert Battey, J. T. Johnson, J. G. Thomas, C. H. Hall, E. Fitzgerald, George F. Cooper, T. S. Hopkins, S. G. Hawkins, J. W. Bailey, E. L. Connelly, A. W. Calhoun, V. H. Tailliaferro, E. W. Alfriend, E. K. Boseman, A. W. Griggs, Thomas H. Kennan, A. B. Calhoun, W. O'Daniel, G. G. Crawford, W. S. Kendrick, F. A. Stanford, W. B. Wells, E. H. Richardson, J. T. Slaughter, John S. Coleman, M. G. Hatch, E. H. W. Hunter, John L. Hamilton, R. J. Nunn, W. H. Hall, P. L. Hillsman.

After passing the usual complimentary resolutions in respect to the hospitalities of the profession and citizens of Atlanta, the Association adjourned to meet in Athens, Georgia, the 3d Wednesday in April, 1883.

